# COVID-19 in multiple sclerosis: The Dutch experience

**DOI:** 10.1177/1352458520942198

**Published:** 2020-07-14

**Authors:** Floor C Loonstra, Elske Hoitsma, Zoé LE van Kempen, Joep Killestein, Jop P Mostert

**Affiliations:** Department of Neurology, Amsterdam Neuroscience, MS Center Amsterdam, Amsterdam University Medical Centers, VU University Medical Center, Amsterdam, The Netherlands; Department of Neurology, Alrijne Hospital, Leiden, The Netherlands; Department of Neurology, Amsterdam Neuroscience, MS Center Amsterdam, Amsterdam University Medical Centers, VU University Medical Center, Amsterdam, The Netherlands; Department of Neurology, Amsterdam Neuroscience, MS Center Amsterdam, Amsterdam University Medical Centers, VU University Medical Center, Amsterdam, The Netherlands; Department of Neurology, Rijnstate Hospital, Arnhem, The Netherlands

**Keywords:** Multiple sclerosis, COVID-19, disease-modifying treatment, lymphocytes

## Abstract

Here, we provide an extensive overview of all reported COVID-19 cases in multiple sclerosis (MS) patients in the Netherlands between 27 February and 9 June 2020, gathered by the Dutch MS Taskforce of the Netherlands Society of Neurology. A total of 86 MS patients were reported, 43 of whom tested positive for COVID-19. Of 43 patients who tested positive, 22 patients were hospitalized. Three intensive care unit (ICU) admissions and four deaths were reported. Our findings show no apparent difference in disease-modifying treatment (DMT) use and COVID-19 disease course in Dutch MS patients. In addition, a clear link between low lymphocyte count and severe disease was not observed.

## Introduction

As the severe acute respiratory syndrome coronavirus 2 (SARS-CoV-2) has spread rapidly around the world, the need for reliable information about the susceptibility to severe COVID-19 disease in multiple sclerosis (MS) patients is becoming increasingly urgent. The effect of disease-modifying treatment (DMT) on COVID-19 disease course is crucial to the therapeutic management, since MS patients with certain treatments are considered as at-risk populations. Consequently, this has led to alterations of immunomodulatory treatment strategies or treatment gaps. Paradoxically, it has been hypothesized that certain immunomodulatory treatments have a beneficial effect on COVID-19 infection.^1^

Although emerging data do not suggest an unfavorable course of COVID-19 disease in patients with MS, data to guide the management and clinical decision making of MS patients during the COVID-19 pandemic are limited.^1–6^ Therefore, we provide an extensive overview of all reported COVID-19 cases in MS patients in the Netherlands, gathered by the Dutch MS Taskforce of the Netherlands Society of Neurology.

## Methods

After the first patient was confirmed in the Netherlands on 27 February, Dutch MS neurologists were requested to report confirmed or suspected COVID-19 MS patients. Data on demographics, MS type, Expanded Disability Status Scale (EDSS), DMT, lymphocyte count, comorbidity, hospital (intensive care) admission, outcome, COVID-19 test confirmation, and level of suspicion were obtained. Descriptive statistics were used to present the data.

## Results

Between 27 February and 9 June 2020, 86 MS patients were reported (Table 1), 43 of whom tested positive. In total, 37 patients had a positive polymerase chain reaction (PCR) on swabs, 4 patients were tested positive based on pathognomonic computed tomography (CT) findings, and in 2 patients antibodies against SARS-CoV-2 were detected by enzyme-linked immunosorbent assay (ELISA). Forty-three patients were suspected of COVID-19, but were not tested according to national regulations.

**Table 1.** Clinical and demographic characteristics of suspected and confirmed COVID-19 MS patients.

	COVID-19 positive			COVID-19 suspected *N* = 43	Total *N* = 86
	Hospitalized *N* = 22	Non-hospitalized *N* = 21	All *N* = 43		
Sex (*n*, %)					
Female	12 (55)	16 (76)	28 (65)	32 (74)	60 (70)
Male	10 (45)	5 (24)	15 (35)	11 (26)	26 (30)
Age (mean, range)	51.7 (39–71)	44.5 (27–71)	48.2 (27–71)	42.7 (20–71)	45.5 (20–71)
MS type (*n*)					
RRMS	12	18	30	39	69
SPMS	6	1	7	2	9
PPMS	3	1	4	1	5
Baló’s MS	0	0	0	1	1
Missing	1	1	2	0	2
EDSS (median, range)	3.5 (1–8)	2.5 (0.5–7)	3 (0.5–8)	2 (0–6.5)	3 (0–8)
Disease-modifying treatment (*n*)					
Dimethyl fumarate	2	6	8	10	18
Glatiramer acetate	1	0	1	3	4
Interferon-beta	1	1	2	3	5
Teriflunomide	1	2	3	2	5
Alemtuzumab	0	0	0	1	1
Fingolimod	2	5	7	8	15
Natalizumab	0	3	3	2	5
Ocrelizumab	6	2	8	11	19
Stem cell therapy^a^	1	0	1	0	1
Immunoglobulin	1	0	1	0	1
No disease-modifying treatment	7	2	9	3	12
Comorbidity^b^	10	4	14	9	23
Hospital admission	22	0	22	0	22
Intensive care unit admission^c^	3	0	3	0	3
Death^d^	4	0	4	0	4

MS: multiple sclerosis; RRMS: relapsing-remitting multiple sclerosis; SPMS: secondary-progressive multiple sclerosis; PPMS: primary-progressive multiple sclerosis; EDSS: Expanded Disability Status Scale; COPD: chronic obstructive pulmonary disease; DMT: disease-modifying treatment.

a One patient who had received autologous stem cell transplantation was hospitalized due to social indication (untenable home situation). Female, aged 41, RRMS, EDSS 7.5, no comorbidity.

b Reported comorbidities: asthma, COPD, obesity, cardiovascular disease, diabetes, malignancy, rheumatic disease, hypothyroidism, and chronic liver disease.

c Patient 1: female, aged 56, RRMS, EDSS 4.0, no comorbidity. Intubation was not required. Patient 2: female, aged 43, RRMS, EDSS 3.0, with obesity, required intubation. Patient 3: male, aged 53, RRMS, EDSS 1.0, required ventilation for 24 days.

d Four deaths were reported. Patient 1: male, aged 57, EDSS 7, no DMT, with asthma and hypertension, refused intensive care unit (ICU) admission. Patient 2: male, aged 59, PPMS, EDSS 4, no DMT, with obesity, no intubation and ICU admission due to fulminant disease. Patient 3: male, aged 59, SPMS, EDSS 5.5, ocrelizumab, COPD GOLD grade 2, refused ICU admission. Patient 4: female, aged 42, RRMS, estimated EDSS 6.0 and severe cognitive impairment, fingolimod, history of struma treated with radioiodine, refused ICU admission.

Of 43 patients who tested positive, 22 patients were hospitalized. The 21 patients who did not require hospitalization were considered to have a mild COVID-19 infection. Hospitalized patients were older, relatively more often male, were more often of the secondary-progressive multiple sclerosis (SPMS) subtype, had a higher EDSS score, and had more comorbidity, compared to the non-hospitalized patients (Table 1).

Three patients were admitted at the intensive care unit (ICU), two using ocrelizumab and one using dimethyl fumarate. In addition, four deaths were reported: one patient with obesity and EDSS 4.0 without DMT, one patient with EDSS 7.0 and asthma without DMT, one patient with EDSS 6.0 and chronic obstructive pulmonary disease (COPD) Global Initiative for Chronic Obstructive Lung Disease (GOLD) grade 2 using ocrelizumab, and one patient with severe cognitive impairment using fingolimod. The first patient had a fulminant disease course and died before he could be intubated. The latter three patients deliberately decided not to be admitted at the ICU.

**Figure 1.**
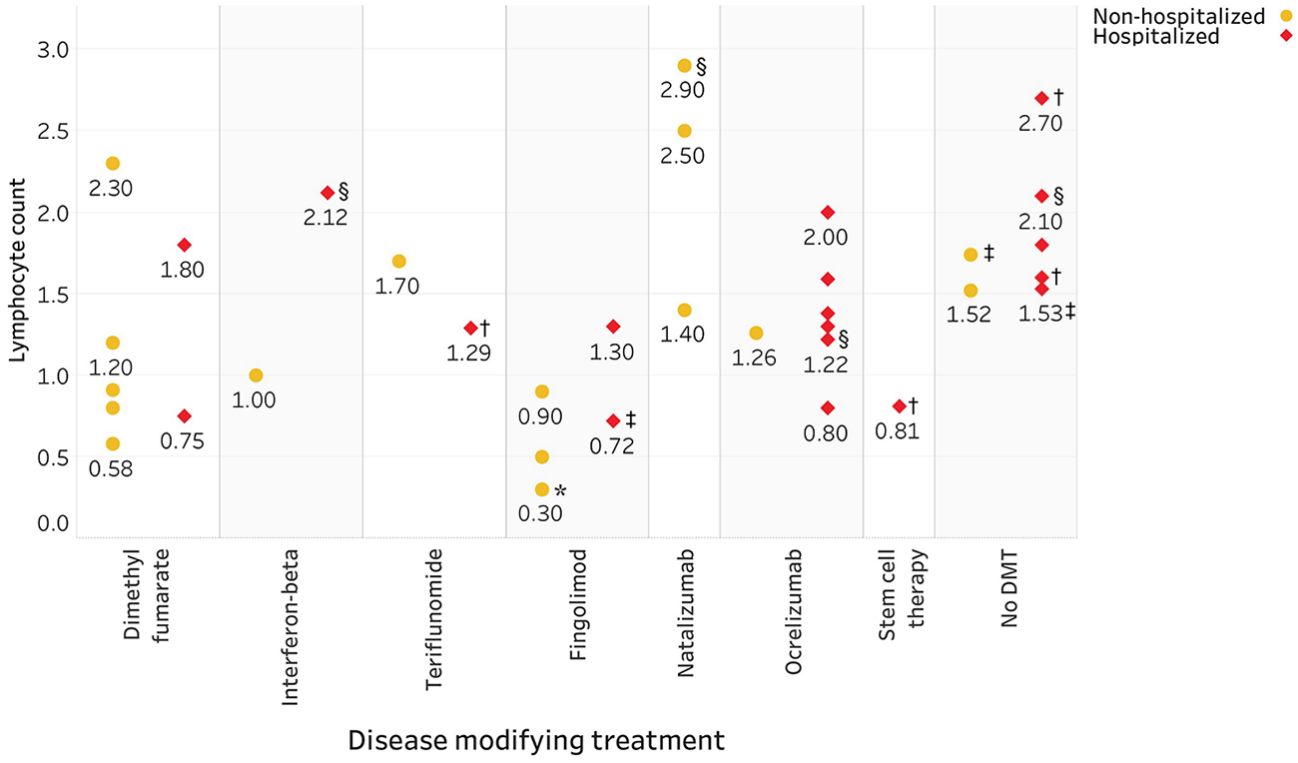
Lymphocyte count in COVID-19-confirmed MS patients (*n* = 36 available), categorized according to disease-modifying therapy (DMT) and hospitalization. All values are prior to COVID-19 infection, unless otherwise specified. *Three patients using fingolimod had a lymphocyte count of 0.3 X 109/L, none of whom required hospitalization. ^†^Lymphocyte count at the time of hospital admission. ^‡^<6 months between last lymphocyte count and COVID-19 infection. ^§^Timing of lymphocyte count is missing.

Importantly, there was no association between severity of COVID-19 and low lymphocyte count (Figure 1). One patient who received autologous stem cell transplantation 5 months before symptom onset had mild COVID-19 disease and good recovery. Two months before COVID-19 symptom onset, the lymphocyte count was 1.42 × 10^9^/L (reference range: 1.00–3.50 × 10^9^/L) and the neutrophil count 3.0 × 10^9^/L (reference range: 1.50–7.50 × 10^9^/L). At the first onset day, characterized by fever (38.8°C), malaise, muscle aches and mild dyspnea, a lymphocyte count of 0.81 × 10^9^/L, and a neutrophil count of 0.57 × 10^9^/L were observed. Based on clinical parameters, there was no need for hospital admission: supplemental oxygen was not required and the patient presented with mild symptoms. However, the patient was hospitalized due to social indication. Due to neutropenic fever, most likely caused by the Corona virus and incomplete bone marrow recovery after stem cell transplantation, levofloxacin and filgrastim were administered at the second day of admission until neutrophil recovery to 1 × 10^9^/L. After 8 days, the patient was symptom-free except for a sore throat, with a neutrophil count of 7.95 × 10^9^/L and lymphocyte count of 2.68 × 10^9^/L.

## Discussion

The possible negative effect of immunomodulatory treatment in MS patients with SARS-CoV-2 infections is a major concern during the pandemic. Although our findings should be interpreted with caution in this small cohort, we did not see a trend of a worse outcome in MS patients on DMT in general. Known risk factors for severe COVID-19 disease such as male sex, comorbidity, and age could be confirmed.^7^

In addition, a clear link between low lymphocyte count and severe disease was not observed. This is of importance as some neurologists advise dose reduction or discontinuing treatment in lymphopenic patients.^1,3^ To date, data on lymphocytes and DMT use in MS patients are lacking.

Compared to current evidence, a relatively high proportion of patients using ocrelizumab were observed in our cohort, of which the vast majority of the positively tested was hospitalized.^2,4^ However, this may be attributed to indication bias and/or reporting bias. Overall, our findings show no apparent difference in DMT use and COVID-19 disease course in Dutch MS patients, which is similar to that reported in an Italian cohort.^2^

Even though our data are reassuring, long-term data acquisition is crucial to gain more knowledge about the potentially protective or harmful nature of immunosuppressive agents in COVID-19 disease, risk factors associated with severe COVID-19, and antibody formation in MS patients.
